# Computational mechanistic studies of ruthenium catalysed methanol dehydrogenation†

**DOI:** 10.1039/d1dt04168a

**Published:** 2022-01-19

**Authors:** Felix J. de Zwart, Vivek Sinha, Monica Trincado, Hansjörg Grützmacher, Bas de Bruin

**Affiliations:** Van ‘t Hoff Institute for Molecular Sciences (HIMS) Science Park 904 1098 XH Amsterdam The Netherlands b.debruin@uva.nl; Inorganic Systems Engineering, Department of Chemical Engineering, Delft University of Technology Van der Maasweg 9 Delft 2629 HZ The Netherlands v.sinha@tudelft.nl; Laboratory of Inorganic Chemistry, Department of Chemistry and Applied Biosciences, ETH Zurich Vladimir-Prelog-Weg 1-5/10 8093 Zurich Switzerland

## Abstract

Homogeneous ruthenium catalysed methanol dehydrogenation could become a key reaction for hydrogen production in liquid fuel cells. In order to improve existing catalytic systems, mechanistic insight is paramount in directing future studies. Herein, we describe what computational mechanistic research has taught us so far about ruthenium catalysed dehydrogenation reactions. In general, two mechanistic pathways can be operative in these reactions: a metal-centered or a metal–ligand cooperative (Noyori–Morris type) minimum energy reaction pathway (MERP). Discerning between these mechanisms on the basis of computational studies has proven to be highly input dependent, and to circumvent pitfalls it is important to consider several factors, such as solvent effects, metal–ligand cooperativity, alternative geometries, and complex electronic structures of metal centres. This Frontiers article summarizes the reported computational research performed on ruthenium catalyzed dehydrogenation reactions performed in the past decade, and serves as a guide for future research.

## Abstract 2

This Frontiers article summarizes the reported computational research performed on ruthenium catalyzed methanol dehydrogenation reactions performed in the past decade, and serves as a guide for future research.

## Graphical abstract

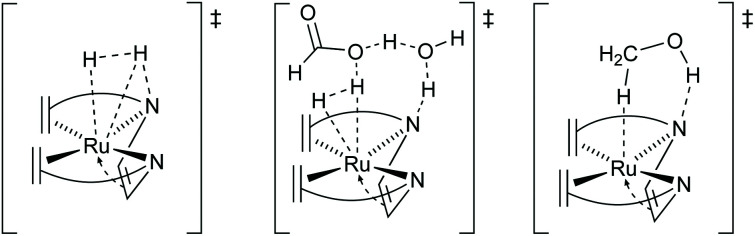

Storage and release of hydrogen in/from stable liquids is a promising technology for *on-demand* application of renewable energy. Hydrogen produced from electrolysis of water using renewable energy sources such as wind and solar can be stored in stable liquid organic compounds such as methanol (or formic acid) by direct reduction of carbon dioxide, which can be obtained from atmospheric carbon capture technology.^1^ When needed, hydrogen stored in methanol can be regenerated by catalytic systems and fed into hydrogen fuels cells.^2^ The clean conversion of methanol–water mixtures into carbon dioxide and high quality hydrogen gas has been intensively investigated in the last decade due to promising applications in energy storage.^3^ Methanol is a harmless, easy-to-store, water-soluble fuel that can be produced industrially from renewable resources.^4^ Methanol–water mixtures have a hydrogen content of 12.0 wt% which can be released through steam reforming of methanol according to the following reactions.^5^

1 H_3_COH + H_2_O → HCOOH + 2H_2_ Δ*H*_r_ = 53.3 kJ mol^−1^

2 HCOOH → CO_2_ + H_2_ Δ*H*_r_ = −14.5 kJ mol^−1^

In eqn (1), methanol is coupled to water into formic acid and two equivalents of hydrogen are released. As this involves the use of water as an oxygen transfer reagent, this reaction is endothermic by 53.3 kJ mol^−1^. Formic acid can be converted further to carbon dioxide according to eqn (2), releasing another equivalent of hydrogen which is an exothermic reaction by −14.5 kJ mol^−1^. The overall reaction is endothermic, but the reaction is driven by entropy (release of H_2_ gas drives the reaction forward). Both homogeneous and heterogenous catalysts have been developed for the methanol dehydrogenation reaction. The heterogeneous catalysts capable of producing hydrogen from methanol require high temperatures (200–300 °C) and produce carbon monoxide which can be poisonous to the catalysts. Selected examples of homogeneous ruthenium-based catalysts discussed in this article are depicted below in Fig. 1.^6–9^ For homogeneous catalysis, two landmark articles were almost simultaneously published in 2013 by the groups of Beller and Grützmacher (Fig. 1).^6,7^ Both catalytic systems contain ruthenium complexes and are capable of releasing the entire hydrogen content (12.0 wt%) of a 1 : 1 methanol–water mixture into CO_2_ and H_2_ at temperatures below 100 °C. The formation of CO was not detected. In the following years, various articles were published to understand the principles that underlie this unique reactivity and high selectivity.^10–14^ More active and even catalysts with earth-abundant metals from the fourth period have been reported since then (such as Fe(PNP) in Fig. 1), and have been thoroughly described in various reviews.^15–18^ However, the ruthenium systems are the ones which are best investigated and can thus aid the design of new catalysts. In this *Frontiers* article, computational investigations of possible mechanisms for ruthenium catalysed methanol dehydrogenations are described together with the possible pitfalls within computational chemistry, with a focus on lessons learned from the [Ru(trop_2_dad)] system. Key findings from this line of research are: (a) The electronic structures of ruthenium complexes are intricate and can show significant multireference character depending on the substrate. (b) Metal–ligand cooperativity (MLC) is a promising design strategy for active, selective and additive-free catalysts. (c) The p*K*_a_ of the ligand and hydricity of metal centre are key descriptors of catalytic activity, determine the mechanism and explain the dependence on external base. (d) Explicit solvent effects are important in mechanistic studies of these systems.

**Fig. 1:**
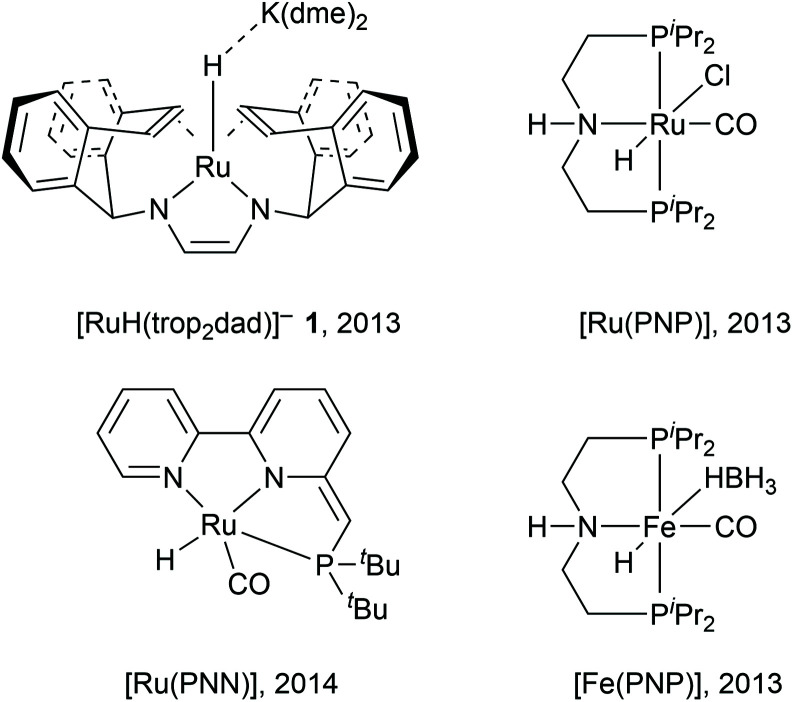
Selected examples of homogeneous catalysts capable of methanol dehydrogenation.

## What does aqueous methanol dehydrogenation entail?

**Scheme 1:**
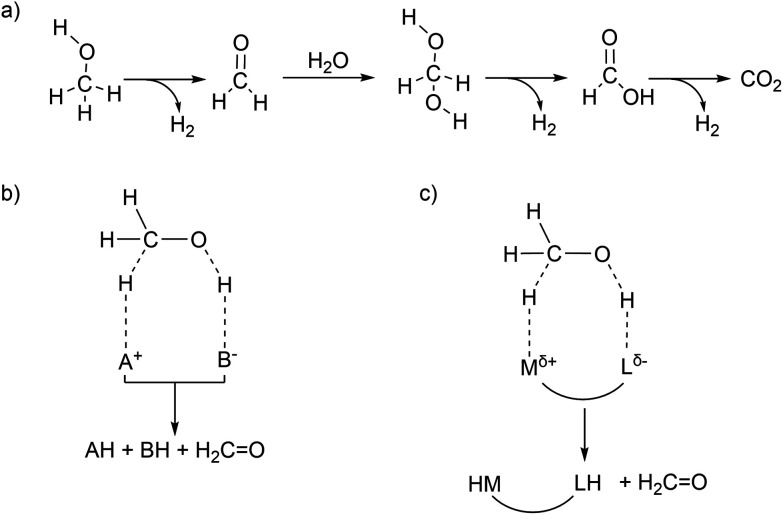
(a) Proposed steps in stepwise dehydrogenation of a methanol–water mixture to produce CO_2_ and 3 eq. of H_2_. (b) Strategy for heterolytic dehydrogenation of methanol enabled *via* a Lewis acid (A^+^) and a Brønsted base (B^−^). (c) Metal–ligand cooperative design strategy to dehydrogenate methanol where the metal acts as a Lewis acid and the ligand acts as a Brønsted base.

Hydrogen production from methanol is proposed to undergo a series of elementary steps proceeding *via* formaldehyde, methanediol, and formic acid intermediates, producing CO_2_ and 3 equivalents of H_2_. Strategically, methanol can be dehydrogenated by using a Lewis acid – Brønsted base pair: the Lewis acid to accept a hydride and the Brønsted base to accept a proton (Scheme 1b). Another possible strategy for the dehydrogenation involves a hydrogen atom transfer (HAT) mechanism, *via* radical intermediates, or *via* electrochemical pathways involving concerted/step-wise H^+^/e^−^ transfer steps. In the present article our focus will be exclusively on a (Lewis) acid–base enabled heterolytic mechanism. The dehydrogenation of methanol yields in the first step formaldehyde (which in presence of water is further stepwise dehydrogenated, Scheme 1a), a metal hydride complex MH, and the conjugate acid LH. In order to produce hydrogen in a catalytic manner, one needs to regenerate the (Lewis) acid–base pair. Ideally the conjugate acid LH should effectively protonate the metal hydride MH to produce H_2_, thereby regenerating the (Lewis) acid–base pair which can be used in the next turnover (Scheme 1b). Combining the Lewis acid and the Brønsted base units in a single complex can enable efficient catalysis without requiring external base additives (Scheme 1c). This design constitutes a metal–ligand cooperative catalysis where the metal and ligand collaborate to catalytically dehydrogenate methanol and produce hydrogen. Importantly, this cooperative catalysis involves a mechanism in which the L/L–H pair is chemically non-innocent, meaning that L/L–H are reversibly chemically transformed analogous to metal–ligand bifunctional catalysis in hydrogenation reactions.^19–21^ Related but distinct mechanisms have been proposed in some hydrogenation reactions (the microscopic reverse of dehydrogenation reactions) in which the ligand is chemically innocent but assists by hydrogen bonding interactions in the catalytic conversion. Here too mechanistic insight has led to improved catalyst design.^22–24^ Various low valent Ru complexes (Fig. 1) are excellent catalysts for efficient aqueous methanol dehydrogenation.^6,7^ A key difference between the complexes [RuH(trop_2_dad)]^−^ and [Ru(PNP)] was that the latter optimally functioned at high (almost stoichiometric) base concentrations, while the [RuH(trop_2_dad)]^−^ complex could catalyse the reaction without any acid or base additives. Several other catalytic systems were reported subsequently, but most of them require high alkaline conditions, or other additives such as an external Lewis acid. Herein, we will describe an overview of our mechanistic investigations for which we used density functional theory (DFT) and *ab initio* molecular dynamics (AIMD) simulations. These calculations are supported by various experiments. In combination they may allow to track down some inherent design principles for catalytic systems that allow aqueous methanol dehydrogenation.

## Methanol dehydrogenation by [Ru(trop_2_dad)]

**Fig. 2:**
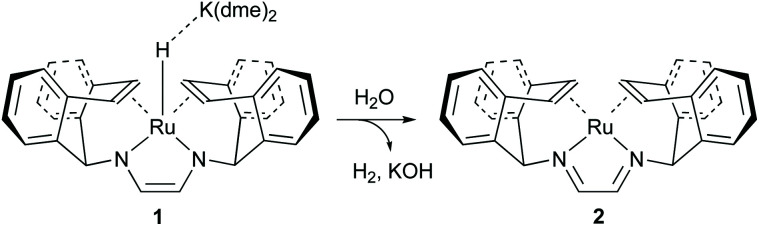
*In situ* formation of neutral [Ru(trop_2_dad)] complex 2 by protonation of the precursor hydride complex 1 (trop_2_dad = 1,4-bis(5*H*-dibenzo[*a*,*d*]cyclohepten-5-yl)-1,4-diazabuta-1,3-diene).

**Fig. 3:**
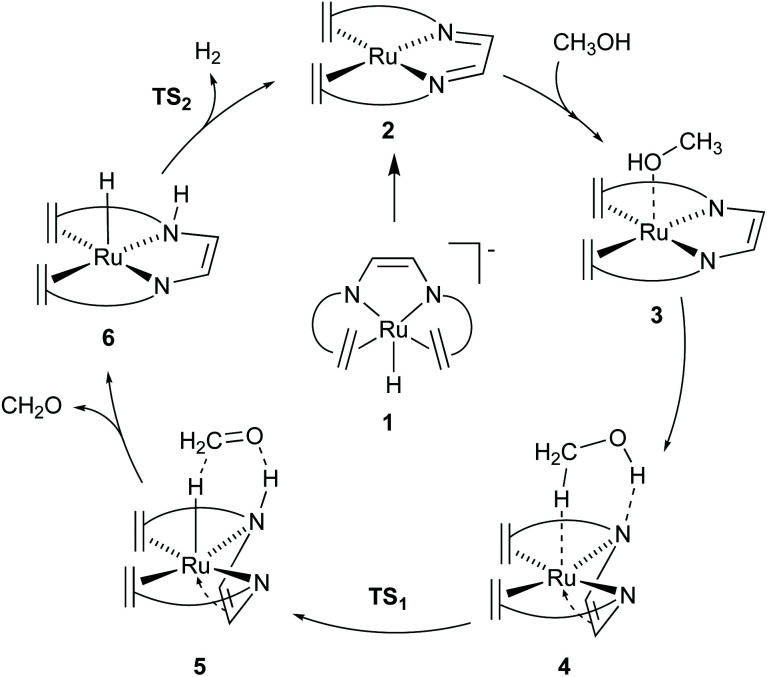
Catalytic cycle for methanol dehydrogenation by [Ru(trop_2_dad)]. Ligand shown as simplified structure, the full structure is shown in Fig. 2.

The [Ru(trop_2_dad)] complex is one example of an efficient ruthenium catalyst for aqueous methanol dehydrogenation.^7^ This work was inspired by previous work on alcohol dehydrogenation with catalysts based on other metal–olefin complexes containing cooperative basic sites.^25^ The ruthenium catalyst is easily synthesized in two reaction steps to the hydride complex 1 which is active in the additive-free methanol dehydrogenation at ambient pressure (Fig. 2). The coordinated diazabutadiene moiety is redox and chemically non-innocent and can reversibly store two equivalents of hydrogen.^26^ When 0.5 mol% of catalyst 1 was added to a methanol–THF–water mixture heated to 90 °C, 80% conversion of methanol to CO_2_ was achieved within 10 hours. Based on *in situ* NMR studies, the ruthenium hydride was proposed to react with water to form the highly reactive complex 2 (Fig. 2). No addition of base was required and methanol dehydrogenation proceeded selectively without the formation of detectable amounts of carbon monoxide. In order to corroborate the formation of formic acid as an intermediate step in methanol dehydrogenation, as shown in Scheme 1a, the [Ru(trop_2_dad)] catalyst was also used for formic acid dehydrogenation. When 0.01 mol% of the hydride complex 1 was added to pure formic acid in dioxane at 90 °C, initial TOFs of up to 24 000 h^−1^ were achieved. The currently accepted catalytic cycle for methanol dehydrogenation catalysed by this complex is shown in Fig. 3. Hydride complex 1 undergoes protonation to form the catalytically active species 2. A substrate molecule can then coordinate to this complex (methanol, methanediol or formic acid), after which the complex rearranges to form 4 with a π-coordinated dad ligand moiety making the N-donor centers in the ligand more basic.^26^ Dihydrogen transfer then occurs in a concerted fashion to form adduct 5, which after formaldehyde decoordination forms complex 6. This complex can then undergo either solvent-assisted, formic acid-assisted, or unassisted dihydrogen release to reform starting complex 2. The [Ru(trop_2_dad)] catalyst system was also used for aqueous formaldehyde dehydrogenation.^27^ The release of hydrogen from formaldehyde/water mixtures (formalin) is exothermic thus allowing the reaction to be performed under milder conditions than required for methanol dehydrogenation. The catalytic system containing [Ru(trop_2_dad)] is active in formalin dehydrogenation with yields up to 90% of H_2_ and TOF_50_ values higher than 20 000 h^−1^ under basic conditions. DFT investigations revealed hydride elimination of methanediol and formic acid to be the key steps in formalin dehydrogenation, with the ruthenium centre playing an active role in catalysis. A detailed understanding of the elementary steps involved is necessary for the development of new and improved catalysts, and thus an in-depth DFT study was performed to study the mechanism of methanol dehydrogenation by [Ru(trop_2_dad)].

## Electronic structure and reactivity of [Ru(trop_2_dad)]

**Fig. 4:**
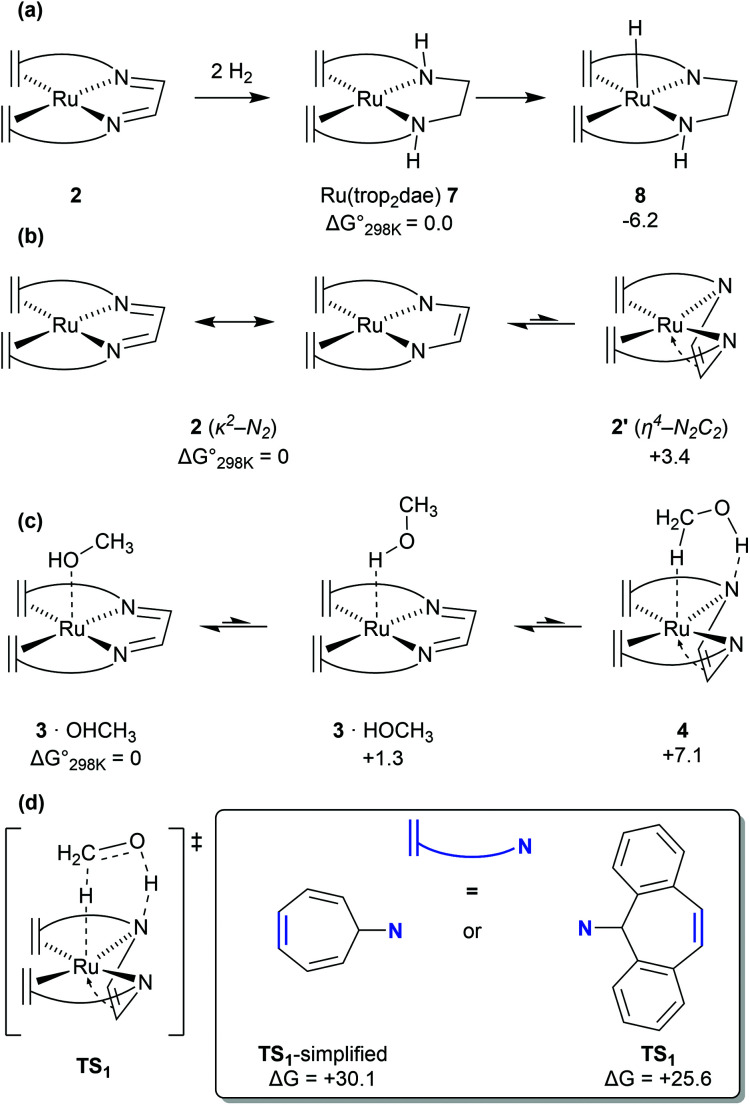
(a) Ligand based hydrogenation of [Ru(trop_2_dad)] to form [Ru(trop_2_dae)]. (b) Resonance forms of [Ru(trop_2_dad)] and coordination modes of the ligand. (c) Rearrangement of methanol (and the ligand) on complex 3 to form pre-activated complex 4. (d) Activated complex at TS_1_ (simplified and full model).

DFT calculations, supported by wavefunction based multireference CASSCF calculations revealed that complex 2 and its adducts with THF—which could be isolated and structurally characterized by X-ray diffraction methods—or water have a multireference character in their wavefunctions.^10^ While the closed-shell singlet state wavefunction dominates, a significant (8–16%) open-shell singlet character corresponding to a [d^7^-Ru^I^(L)(trop_2_dad˙^−^)] electronic structure contribution is also present. Such an open-shell character was found to open new reaction channels featuring both metal-centred, and ligand-centred reactivity. DFT calculations further suggested that during methanol dehydrogenation the open-shell character is suppressed as a substrate (methanol) approached complex 2, to form weak adducts such as 3·OHMe or 3·HOMe (Fig. 4c). DFT calculations on the neutral [Ru(trop_2_)dad] complex 2 revealed that the N_DAD_ moiety primarily existed in the diimine form (Fig. 4b). Potential energy surface scans revealed that methanol interacted favourably with Ru centre in complex 2*via* metal–proton interaction, and did not preferentially form hydrogen bonds with the N_DAD_ moiety. However, DFT calculations further revealed that the dad moiety in 2 is flexible and can undergo a slightly endergonic (3.4 kcal mol^−1^) π-coordination with Ru which is hindered by only a very small activation barrier of 3.8 kcal mol^−1^ to form complex 2′ (Fig. 4b).^13^ This change of the dad coordination mode from κ^2^-N_2_ to η^4^-N_2_C_2_ leads to an enhancement of the Brønsted basicity of the nitrogen centres of the DAD moiety which consequently do interact now with the OH group in MeOH *via* H-bonding to give the methanol adduct 4 (Fig. 4c). Because the relative energy differences between 3·OHMe, 3·HOMe, and 4 are very small, it can be assumed that these complexes coexist in equilibrium. Through natural population analysis of the methanol adducts of [Ru(trop_2_dad)] 3 and 4 it was shown that in the π-complex the net negative natural charge is 0.10*e* higher on the nitrogen atom, highlighting the increase in basicity. This pre-organises the methanol molecule for subsequent hydride transfer, lowering the TS barrier through active ligand cooperation.

## Mechanism of methanol dehydrogenation

The mechanism for dehydrogenation of methanol consists of two main steps: (a) transfer of a hydride and a proton from methanol to the catalyst (assuming a heterolytic mechanism); and (b) release of dihydrogen to regenerate the catalyst complex. Quite interestingly complex 2 can also undergo ligand hydrogenation by two equivalents of hydrogen to form the [Ru(trop_2_dae)] complex 7 (Fig. 4a). This complex was observed experimentally and was originally thought to be involved as a reversible store of hydrogen equivalents in the catalytic cycle. However, DFT calculations by Li & Hall and our group, clearly suggested that the required transition state barriers to dehydrogenate the ligand backbone in the [Ru(trop_2_dae)] complex were very high.^12,13^ However, DFT calculations revealed that the [Ru^0^(trop_2_dae)] complex can undergo a proton shift from the N_DAD_–H moiety to the metal centre to generate the Ru^II^–hydride complex 8 (Fig. 4a).^27^ This proton shift also generates the catalytically relevant Ru^II^–N^−^ moiety, which is key in the dehydrogenation reaction of methanol/methanediol/formic acid. In fact, when the catalytic dehydrogenation of aqueous formaldehyde was performed using [Ru(trop_2_dae)] complex 7, it showed catalytic activity and yields of H_2_ were obtained which are comparable to the ones achieved with the anionic [Ru(H)(trop_2_dad)]^−^ complex.^27^ Therefore, the [Ru(trop_2_dae)] complex acts as an independent catalyst that forms *in situ* from the [Ru(trop_2_dad)] complex.

### Importance of full-atom models

**Fig. 5:**
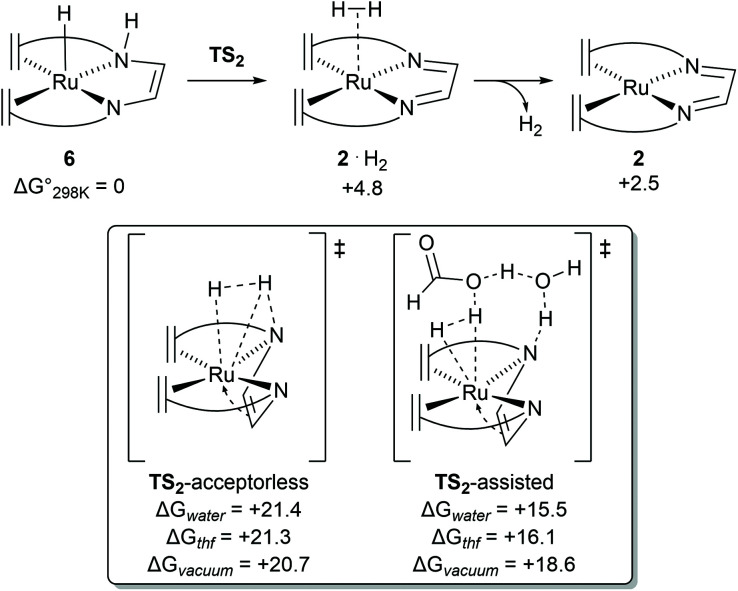
Transition state barriers for dihydrogen release from complex 6 in the catalytic dehydrogenation of methanol using [Ru(trop_2_dad)] complex 2.

Previous DFT studies did not identify the η^4^-N_2_C_2_ bound π-complex 4 as a likely intermediate. This was presumably due to the use of simplified atom models which underestimate the stability of the π-complex.^12^ In order to speed up calculations, the tropylium moiety is often simplified as a cycloheptatriene unit. When the transition state for dihydrogen transfer was calculated with both the full-atom and simplified models, it was shown that the simplified model provides a far higher barrier at TS1 = +30.1 kcal mol^−1^ (Fig. 4d), whereas the full atom model gave a barrier of +25.6 kcal mol^−1^.^13^ The previously investigated formic acid dehydrogenation was revisited with this knowledge, and it was also shown that η^4^-coordination of the N_2_C_2_-backbone lowers the hydride transfer barrier by 4.3 kcal mol^−1^ when a full-atom model is used. The release of dihydrogen from the ruthenium complex was shown to be influenced by explicit solvation (Fig. 5). In the unassisted reaction, the barrier at TS2 is +21.4 kcal mol^−1^. When a formic acid–water complex is added to assist in the release of dihydrogen, the transition state barrier drops by 5.9 kcal mol^−1^. In the early stages of catalysis, hydrogen release could proceed unassisted, but at higher conversions hydrogen release is likely to proceed through the assisted pathway. The κ^2^-N_2_ to η^4^-N_2_C_2_ rearrangement proved crucial for alcohol activation and was shown to be important for formic acid dehydrogenation as well.^13^ The increased basicity on the ligand nitrogen atoms, and the increased Lewis acidity on the metal allows for a metal–ligand cooperative Noyori–Morris type mechanism where dihydrogen is transferred to ruthenium and the ligand in a concerted manner. This effect is most pronounced when full-atom models were used and as such this investigation highlights the perils of using simplified models for calculations.

### Solvent effects

Dehydrogenation of methanol (and methanediol/formic acid) proceeds *via* hydride and proton transfer states. The underlying transition states and intermediates involve charge polarized states where a C−H, O−H or N−H bond is being formed or cleaved. Solvent effects are therefore expected to play an important role in the mechanism, *via* explicit interaction such as reactive adsorption and hydrogen bonding, and implicit effects through stabilization of charge polarized states. In case of the catalytic system with complex 2 or the hydrogenated complexes 7 and 8 (formed *in situ*) act as the primary activated catalyst complexes. For complex 2, DFT calculations revealed that the trop_2_dad ligand creates a hydrophobic environment around the Ru–N bonds, and explicit solvent effects were not found to play a direct and important role in the hydrogenation of the catalyst. Inclusion of explicit solvent molecules such as water or methanol in the hydrogen release steps of the reaction led to increased activation barriers. Lower barriers were obtained only when both formic acid and water were included as a shell around the catalyst, which lowers the barrier for H_2_ release, probably driven by the higher acidity of formic acid (Fig. 5). DFT calculations performed in vacuum, and with corrections for implicit solvent effects, revealed that the activated complexes at the transition states for hydride transfers were stabilized by 2–4 kcal mol^−1^ in THF when compared to the gas phase. Solvation in water further stabilized these activated complexes by ∼1 kcal mol^−1^. Mechanism for dehydrogenation of calculations with explicit inclusion of the interaction between solvent molecules and intermediates or activated complexes on the methanol-to-formaldehyde reaction pathway following the Noyori–Morris mechanism were performed with complex 2 as possible dehydrogenation catalyst. The possibility that the hydride and proton transfer do not occur in a concerted manner within one activated complex was investigated. However, static DFT calculations on microsolvated complexes did not reveal a mechanism with separate hydride and proton transfer steps. Yet such a mechanistic possibility cannot be completely ruled out and a separate proton transfer step with a very low free energy barrier can precede the hydride transfer step.^28^ The resulting barriers for these processes using calculations in which microsolvated activated complexes were explicitly considered were found to be within 0.2 kcal mol^−1^.^13^ The transition state leading to hydrogen production on the pathway using a solvent continuum model (cosmo) but in which the activated complex is unassisted by explicit solvent molecules suffered from a slight destabilization (∼0.7 kcal mol^−1^) in the solvated phase, while on the reaction pathway assisted by interactions with formic acid and explicit water molecules was stabilized by ∼3 kcal mol^−1^ in water when compared to the gas phase (see Fig. 5). For methanol dehydrogenation *via* complex 2, the solvent can therefore at most play an *assistive role* where it can interact and stabilize intermediates and transition states in the catalytic cycles, or mediate proton transfer steps but does not strongly affect the catalytic mechanism. We therefore concluded that the primary effect of solvent is that it serves as a dielectric medium stabilizing the charge polarized states, and explicit interactions were not found to play a significant role.

**Fig. 6:**
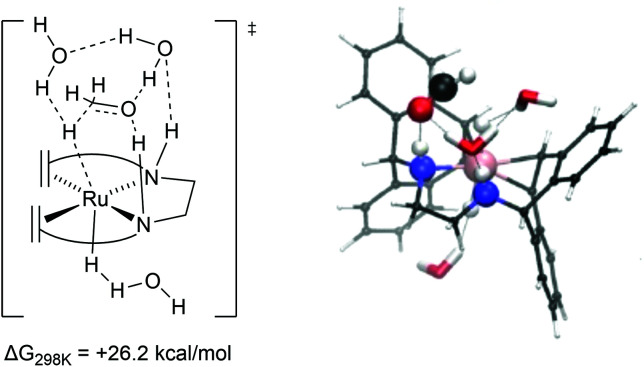
Transition state geometry for C−H activation of methanol by complex 8 obtained from micro-solvation based DFT models along with the computed activation energy barrier (relative to the methoxide adduct in kcal mol^−1^.

In marked contrast to complex 2, strong explicit solvent effects were found to be critical in describing the mechanism of methanol dehydrogenation with complex 8 as catalyst. In the absence of explicit solvent molecules, potential energy surface scans showed that formaldehyde could not be formed by cleaving the C–H bond of methanol at complex 8. This shows that in the gas phase breaking the C–H bond does not lead to a stable product *via* an accessible transition state. As such, a DFT–MD and microsolvated static DFT approach was chosen to implement explicit solvent molecules in the calculations.^14^ For the rate-limiting step, which is C–H activation of methoxide to formaldehyde, a computed barrier of +26.2 kcal mol^−1^ was found by DFT calculations including effects of microsolvation. At this transition state the explicit consideration of solvation of the activated complex plays a crucial role in the mechanism. The anionic oxygen moiety was found to have a strong interaction with the protons on the ligand and the solvent (Fig. 6). Furthermore, the incoming hydride on the metal centre was found to be stabilized by hydrogen bonding interactions with the solvent, stabilizing the transition state and assisting the C–H activation process. A potential energy surface scan and approximate intrinsic reaction coordinate calculations (IRCs) revealed that the explicit interaction of the solvent with the hydride was decisive for cleaving the C–H bond.^13,14^ In this case, the hydride transfer proceeds *via* a metal-centred, solvent-assisted mechanism.

**Fig. 7:**
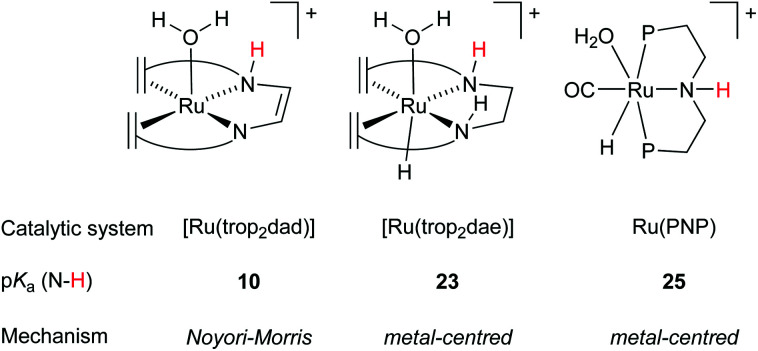
Computed p*K*_a_ of the NH moiety and the mechanism operating for hydride transfer during methanol dehydrogenation.

Another highly active ruthenium system is the one published by Beller *et al.* in 2013, shown in Fig. 1. The well-defined molecular catalyst [Ru(PNP)] shows high activities and achieves a total turnover number of 350 000 over 23 days at 91 °C and full conversion of all available hydrogen atoms in methanol.^6^ However, this system requires strong alkaline conditions to be active. Interestingly, as the catalytic cycle was proposed to operate *via* a mechanism involving metal–ligand cooperation in which the Ru centre is directly bound to an amido unit as internal base there should actually not be additional base or other additives required for catalysis to take place. However, the [Ru(PNP)] systems perform only optimally under highly basic conditions (8 M KOH). As seen before, when catalysis is performed in protic solvent mixtures, explicit and implicit solvent modelling is imperative to gain an accurate picture of the mechanism.^29–34^ Notably, when the [Ru(PNP)] system was subjected to DFT–MD simulations in a water solvent box, the nitrogen atom was spontaneously protonated,^35^ making its participation in a cooperative mechanism in protic solvents rather unlikely. The basicity of the amido moiety in the [Ru(PNP)] complex was further investigated using AIMD simulations, and was found to correspond to a p*K*_a_ of 25 in the resting state (Fig. 7). The p*K*_a_ of the nitrogen centres in the ligand was also studied for other possible species proposed in the catalytic cycle. All calculated p*K*_a_ values are generally high and it can be assumed that they remain protonated during the entire cycle.^36^ Consequently, the previously postulated Noyori-type mechanism is not in agreement with the DFT results and *reversible* protonation of the amido moiety is unlikely to occur under the applied experimental conditions. When the catalytic cycle using [Ru(PNP)] was investigated computationally using explicit solvent effects, the hydride transfer from the coordinated methoxide anion to the metal centre *via* a metal-centred mechanism was found to be rate limiting. This explains the need to use high base concentrations as that will lead to a higher methoxide anion concentration. While the N−H moiety in Ru(PNP) is not actively involved in the hydrogen transfer steps, the structure of the activated complex at the transition state where the hydride is transferred from the methoxide unit to Ru suggests that the N−H moiety may play the role as a directing group by positioning the methoxide (*via* hydrogen bonding) in a proper orientation with respect to the metal center.^19,37^

### Future ligand design and mechanistic descriptors

**Fig. 8:**
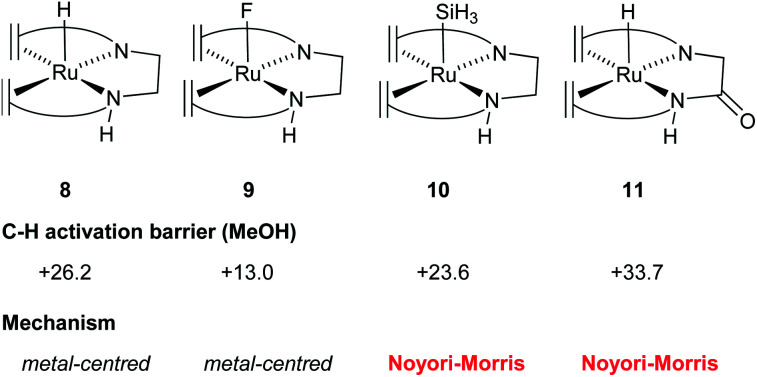
*In silico* suggested examples to tune the activation energy barriers and the reaction mechanism for dehydrogenation of methanol by the [Ru(trop_2_dae)] catalytic system by selective functionalization of the complex.

In order to gain understanding for the various possible mechanisms for methanol dehydrogenation, the p*K*_a_ of the [Ru(trop_2_dad)] system was also investigated. The p*K*_a_ was found to be a good predictor for whether the active complexes in a catalytic dehydrogenation participate in a Noyori–Morris type mechanism or a metal centred (solvent-assisted) mechanism (Fig. 7). Based on these mechanistic insights, it is possible to evaluate the effect of derivatization of existing catalysts on the catalytic performance. Specifically, the hydricity of the metal centre can be enhanced by substituting the trans hydride moiety, and the p*K*_a_ of the ligand can be enhanced by derivatizing the ligand backbone. As such, the C−H activation barrier for derivatives of [RuH(trop_2_dae^−^)] complex 8 was evaluated and the nature of the transition state was investigated. Indeed, when the hydride on the metal centre was substituted by a fluoride, the increased Lewis acidity of the metal centre led to a decrease in the activation energy barrier (see Fig. 8, complex 9). Interestingly, the C−H activation barrier was also lowered when the *trans*-orientated ligand was replaced by an electron rich SiH_3_ group, due to destabilization of the methoxide adduct, resulting in a switch to a Noyori-type mechanism where metal and ligand cooperate (Fig. 8, complex 10). Furthermore, the incorporation of an amide functionality into the ligand backbone was expected to decrease the p*K*_a_ of the NH groups, thereby lowering the activation barrier in the solvent assisted mechanism. Instead, the activation barrier increased to 34 kcal mol^−1^. Furthermore, the mechanism changed from metal-centred to a Noyori-Morris-type mechanism as well (Fig. 8, complex 11). This reflects the importance the p*K*_a_ of the ligand can have on the preferred mechanism, and can guide future ligand design for complexes suitable as efficient methanol dehydrogenation catalysts. Future computational efforts will focus on the understanding of the role of Lewis acid co-catalysts in dehydrogenation reactions. Simple salt additives such as LiBF_4_ or NaCl can strongly promote the catalytic activity but their exact role is currently not fully understood.^38^

## Conclusions

The mechanistic investigations of [Ru(trop_2_dad)] and [Ru(PNP)] catalyst systems provide some important lessons in the design of metal–ligand cooperative catalysts. Modelling studies need to be rigorous about assumptions being made. For example, simplified catalyst model can be helpful in speeding up mechanistic studies but must be carefully tested. Solvent effects can have an important mechanistic role, exclusion of which can render incomplete or even misleading conclusions. The κ^2^-N_2_ to η^4^-N_2_C_2_ rearrangement in the [Ru(trop_2_dad)] complex results in a controlled increase in the basicity of the ligand and acidity of the metal centre. In contrast, the ligand p*K*_a_ is found to be rather high for the [Ru(PNP)] and [Ru(trop_2_dae)] complexes, which hinders metal–ligand cooperativity by inhibiting reversible protonation at the ligand site. The hydricity of the M–H bond formed upon the dehydrogenation of the substrate is an important mechanistic parameter, and together with the p*K*_a_ these parameters determine whether methanol dehydrogenation proceeds through a Noyori–Morris or a metal-centred type mechanism. The availability of metal–ligand cooperativity is clearly not a necessity for catalytic activity, and both the hydricity of M−H moiety and the p*K*_a_ of the L–H moiety needs to be balanced for optimal metal–ligand cooperativity in methanol dehydrogenation catalysis.

## Author contributions

FdZ – Conceptualization, writing – original draft. VS – Conceptualization, writing – review and editing. HG, MT, BdB – Writing – review and editing.

## Conflicts of interest

There are no conflicts to declare.

## Supplementary Material
